# Splice-shifting oligonucleotide (SSO) mediated blocking of an exonic splicing enhancer (ESE) created by the prevalent c.903+469T>C *MTRR* mutation corrects splicing and restores enzyme activity in patient cells

**DOI:** 10.1093/nar/gkv275

**Published:** 2015-04-15

**Authors:** Bruno Palhais, Veronica S. Præstegaard, Rugivan Sabaratnam, Thomas Koed Doktor, Seraina Lutz, Patricie Burda, Terttu Suormala, Matthias Baumgartner, Brian Fowler, Gitte Hoffmann Bruun, Henriette Skovgaard Andersen, Viktor Kožich, Brage Storstein Andresen

**Affiliations:** 1 Department of Biochemistry and Molecular Biology and the Villum Center for Bioanalytical Sciences, University of Southern Denmark, Odense M, Denmark; 2 Division of Metabolism, University Children's Hospital, Zürich, Switzerland; 3 Institute of Inherited Metabolic Disorders, Charles University in Prague-First Faculty of Medicine and General University Hospital, Praha, Czech Republic

## Abstract

The prevalent c.903+469T>C mutation in *MTRR* causes the *cblE* type of homocystinuria by strengthening an SRSF1 binding site in an ESE leading to activation of a pseudoexon. We hypothesized that other splicing regulatory elements (SREs) are also critical for *MTRR* pseudoexon inclusion. We demonstrate that the *MTRR* pseudoexon is on the verge of being recognized and is therefore vulnerable to several point mutations that disrupt a fine-tuned balance between the different SREs. Normally, pseudoexon inclusion is suppressed by a hnRNP A1 binding exonic splicing silencer (ESS). When the c.903+469T>C mutation is present two ESEs abrogate the activity of the ESS and promote pseudoexon inclusion.

Blocking the 3′splice site or the ESEs by SSOs is effective in restoring normal splicing of minigenes and endogenous *MTRR* transcripts in patient cells. By employing an SSO complementary to both ESEs, we were able to rescue MTRR enzymatic activity in patient cells to approximately 50% of that in controls.

We show that several point mutations, individually, can activate a pseudoexon, illustrating that this mechanism can occur more frequently than previously expected. Moreover, we demonstrate that SSO blocking of critical ESEs is a promising strategy to treat the increasing number of activated pseudoexons.

## INTRODUCTION

Expression of protein coding genes in eukaryotes relies on correct splicing of pre-mRNA transcripts. During this process the spliceosome removes intronic sequences from the initial transcripts and joins together the exons to produce a mature mRNA. It is thus crucial for the cell to identify and process exons with high fidelity. Splice site sequences are the major splicing signals recognized by the spliceosomal machinery but due to their degeneracy (1,2) they are not by themselves sufficient for efficient recognition of exons, and *in silico* analysis shows that non-functional copies of splice site sequences are highly abundant in intronic regions (3). Therefore, other *cis*-acting splicing regulatory elements (SRE) are necessary to direct the spliceosomal proteins to the correct splice sites for efficient splicing. Exonic splicing enhancers (ESE) and intronic splicing enhancers (ISE) are sequences commonly bound by proteins of the serine/arginine-rich (SR) family, which stimulate exon inclusion (4). Conversely, members from the heterogeneous nuclear ribonucleoprotein (hnRNP) family bind to exonic splicing silencers (ESS) and intronic splicing silencers (ISS) to repress exon inclusion (5).

Aberrant splicing often causes human diseases and according to the Human Gene Mutation Database (HGMD^®^ Professional Release 2014.4) 14 849 of 163 670 (i.e. about 9.1%) of all reported mutations affect the splicing process. Furthermore, an estimated 25% of the mutations presumed to be missense and nonsense mutations are in fact splicing mutations (6,7). The majority of the reported splicing mutations alter the conserved splice sites at exon–intron junctions. However, in a growing number of cases aberrant splicing results from mutations in positive or negative SREs (3,8–13).

Sequences resembling functional splice sites (pseudosplice-sites) are highly frequent in introns but are rarely used during splicing (3,14). It is believed that this may be due to intrinsic defects in the sequences themselves (14) and the enrichment of splicing silencer motifs (15). When two matching pseudosplice-sites are located close in an intron they define a pseudoexon. Activation of a pseudoexon, so that it is spliced into an mRNA, disrupts gene expression and will often cause disease. All genes harbor pseudoexons, but the number of pseudoexons in our genome is not known and has so far only been loosely estimated based on computational approaches (15,16). Pseudoexon activation has traditionally been regarded as a rare disease mechanism, which requires multiple changes (mutations) to occur (14), but in recent years it has become clear that apparently benign single nucleotide variations can be sufficient to activate pseudoexons and cause disease and the number of reported cases has been increasing (3,17). In most of the reported cases, pseudoexon activation results from single nucleotide changes creating new splice sites or increasing the strength of existing suboptimal splice sites. This is most likely due to the fact that changes involving the splice site sequences are easier to recognize since the consensus motifs are well established. We have recently reported that the most frequent mutation in the methionine synthase reductase (*MTRR*) gene, a deep intronic mutation (c.903+469T>C), creates an SRSF1 binding ESE, which leads to pseudoexon inclusion and causes the cblE type of homocystinuria (18). Similarly, other groups have also reported in other genes (*PCCA*, *GLA*, *FGB*, *CFTR*, *ATM, Col4A5* and *MFGE8*) that single nucleotide changes in introns located outside splice site sequences can cause pseudoexon activation and human disease (19–25). These and other studies convincingly show that an intronic single nucleotide change by affecting splicing regulatory elements outside of the splice sites is sufficient to activate a pseudoexon and cause disease. It can therefore be hypothesized that pseudoexons, like constitutive exons, are regulated by a finely tuned balance between positive and negative splicing regulatory elements and any single nucleotide substitution that changes this balance may lead to pseudoexon activation. Because intronic sequences are typically not examined during routine diagnostic procedures it is likely that the prevalence of pseudoexon activation may thus be underestimated. Additionally, the pseudoexon containing mRNA may be degraded by NMD (26) due to premature termination codons (PTCs) resulting from frame-shifts in the coding sequence. Therefore, it can be a challenge to identify disease-associated mutations caused by pseudoexon activation.

Complementation group cobalamin E (cblE) type of homocystinuria (*cblE*; MIM# 236270) is a rare autosomal recessive disease caused by deficiency of methionine synthase reductase (MTRR; EC 1.16.1.8) (27,28). Methionine synthase (MTR; EC 2.1.1.13) is a cobalamin-dependent enzyme, which remethylates homocysteine to methionine in the remethylation pathway of the methionine cycle and MTRR is required to maintain methylcobalamin (MeCbl) in its reduced form. During its cycle, the cob(I)alamin cofactor of MTR becomes oxidized to cob(II)alamine rendering the enzyme inactive. The dual redox flavoprotein MTRR is then necessary for the reductive activation of MTR. Patients with MTRR deficiency exhibit hyperhomocysteinemia, homocystinuria and often hypomethioninemia, and manifest clinically with failure to thrive, developmental delay megaloblastic anemia and white matter abnormalities (28,29). This severe disease is treated mostly by betaine administration, which supports the remethylation of homocysteine. The *MTRR* gene is composed of 15 exons and 15 disease causing mutations located in different exons were reported (29). The most frequent mutation is a T>C transition located deep within intron 6 (c.903+469T>C, p.S301fsX315) (28,29). We have previously shown that the c.903+469T>C mutation creates an ESE bound by SRSF1, which facilitates the recognition of a weak 5′splice site, leading to pseudoexon inclusion (18).

In recent years, short oligonucleotides have been used to correct splicing defects (30). Splice-shifting oligonucleotides (SSOs) are designed to be complementary to a specific sequence in the pre-mRNA, which regulates the inclusion/skipping of the exon. Modulation of the splicing outcome with SSOs is a promising therapeutic approach because it allows for the correction of disease-causing mutations affecting splicing without the need for gene-replacement (31). Mutations leading to pseudoexon activation often create or strengthen pre-existing splicing signals, such as pseudo-splice sites and SREs, which can be targeted by SSOs to restore normal splicing (17). Targeting the 3′ss or the 5′ss sterically hinders binding of U2AF and U1snRNP, respectively, and may thus inhibit inclusion of the pseudoexon.

In the present study we use splicing reporter minigenes to investigate the pseudoexon activation mechanism in more detail and in particular the role of an additional ESE and an ESS flanking the previously described ESE created by the c.903+469T>C mutation. To explore the possibility of novel therapies for this type of splicing defects we subsequently investigated whether SSOs can correct missplicing and restore enzyme activity in fibroblasts derived from a patient homozygous for the *MTRR* pseudoexon activating mutation.

## MATERIALS AND METHODS

### Minigenes

The *MTRR* minigene (beta-globin variant) has previously been described (18). Mutations in the *MTRR* minigene were introduced by GeneScript Inc. (GenScript, Piscataway, NJ, USA). Wild-type and mutant double stranded DNA oligonucleotides corresponding to 21 nt. of five different pseudoexons were inserted into the alternatively spliced second exon in the RHC-Glo splicing reporter minigene (32) as previously described (33). All constructs were sequenced.

### Splicing analysis of minigenes

HEK293, HeLa or HepG2 cells were plated in 6-well plates so that each well contained 3 × 10^5^ cells. After 24 h of incubation the cells were transiently transfected with 0.8 μg of either the wild-type or the mutant versions of MTRR minigenes using using X-tremeGENE 9 DNA (Roche, Indianapolis, IN, USA). Total RNA was extracted 48 h post-transfection using Isol-RNA Lysis Reagent (AH Diagnostics, Aarhus, Denmark). The RNA was treated with RQ1 RNase-Free DNase (Promega, Madison, WI, USA) and ethanol precipitated afterward. Reverse transcription was performed using the High Capacity cDNA Reverse Transcription Kit (Life Technologies, Thermo Fisher Scientific Inc., Waltham, MA, USA). Polymerase chain reaction (PCR) was performed with forward T3 (5′-aattaaccctcactaaaggg-3′) and reverse T7 (5′-taatacgactcactataggg-3′) plasmid specific primers. Quantification of the PCR products was performed in a Fragment Analyzer instrument (Advanced Analytical Technologies).

### Transfection of HEK293 cells followed by SSO treatment

Approximately 3 × 10^5^ HEK293 cells were plated in 6-well plates. After 24 h the cells were transiently transfected with 0,8 μg of minigene plasmid DNA. 24 h post-transfection the cells were transfected with 20 nM of *MTRR* ESE-SSO (5′-gcugaaucuccuccggccauucuu-3′), *MTRR* 5′SS-SSO (5′-uuccuggcugaccuuaguggacagc-3′), *MTRR* 3′ss-SSO (5′-ucccuaguccuucccugaaguggaa-3′) and a non-targeting SSO using Lipofectamine RNAiMAX transfection reagent (Life Technologies). The SSOs were 2′-OMe-substituted phosphorothioate RNA (DNA Technology, Aarhus, Denmark). Total RNA was extracted and RT-PCR was performed as described above.

### Treatment of patient cells with SSOs

Reverse transfection was performed with 90 nM of each SSO using Lipofectamine RNAiMAX transfection reagent (Life Technologies). Patient and control fibroblast cells were plated in parallel in 6-well plates at a concentration of 15 × 10^4^ cells per well. After 48 h of incubation total RNA was extracted and cDNA synthesis was performed using Superscript VILO Master Mix (Invitrogen Co., Carlsbad, CA, USA). RT-PCR amplicons were separated by gel electrophoresis.

### RNA affinity purification of nuclear proteins

The affinity purification of RNA binding proteins was performed with 3′-biotin coupled RNA oligonucleotides (DNA Technology, Aarhus, Denmark) as previously described (33). The sequences of the RNA oligonucleotides were: WT (5′-ACUAGGGAAGAAUGGCUGGAGGAGA-BIO-3′), MUT (5′-ACUAGGGAAGAAUGGCCGGAGGAGA-BIO-3′), TCGGGA (5′-ACUCGGGAAGAAUGGCUGGAGGAGA-BIO-3′). For each purification 100 pmol of RNA oligonucleotide was coupled to 100 μl of streptavidin-coupled magnetic beads (Invitrogen Co., Carlsbad, CA, USA) and incubated with HeLa nuclear extract (Cilbiotech S.A., Belgium). After washing, bound proteins were investigated by western blotting using a monoclonal mouse antibody toward SRSF1 (AK96) from Zymed Laboratories (Invitrogen Co., Carlsbad, CA, USA) or a monoclonal antibody toward hnRNP A1 (sc-10029) from Santa Cruz Biotechnology (Santa Cruz, CA, USA).

### Fibroblasts

Primary fibroblasts were grown in Dulbecco's Modified Eagle's Medium (DMEM+GlutaMax™, Life Technologies), supplemented with 10% (v/v) foetal calf serum (Life Technologies) and antibiotic-antimycotic mixture (penicillin 10 000 IU/ml, streptomycin 10 000 μg/ml, amphotericin B 25 μg/ml, Life Technologies).

Transformed fibroblasts were obtained by transfecting primary fibroblasts with pRNS1 plasmid containing SV40 DNA sequences (28). Fibroblast from the Czech patient homozygous for the c.903+469T>C mutation (patient 2) in (28) were taken from the repository and used for the studies after obtaining informed written consent of the patient's legal guardians. The study was approved by the Ethics Committee of the General University Hospital, Prague (No 1889/12 S-IV).

### Treatment of fibroblasts with SSOs

Reverse transfection was performed with 90 nM of each SSO using Lipofectamine transfection reagent (Life Technologies). Transformed patient and control fibroblast cells were plated in parallel in 6-well plates at a concentration of 15 × 10^4^ cells per well. After 48 h of incubation total RNA was extracted and cDNA synthesis was performed using Superscript VILO Master Mix (Invitrogen Co., Carlsbad, CA, USA). RT-PCR amplicons were separated by gel electrophoresis.

Transfection of transformed fibroblasts by electroporation was performed with 450 nM of each SSO using a Gene Pulser II electroporator and 0.4 cm gene-pulser cuvettes (Bio-Rad). Total RNA was isolated from the cells 48 h after electroporation for RT-PCR analysis as described above.

### MTRR activity in fibroblasts after SSO transfection

MTRR activity was determined indirectly by metabolically labeling of fibroblasts with [^57^Co]cyanocobalamin and measuring synthesis of MeCbl and AdoCbl, respectively (34,35). Shortly, radioactively labeled medium was added 24 h after lipofection/electroporation and radioactively labeled cobalamins extracted after 3 days incubation followed by HPLC separation and quantitation by gamma-counting. Levels of MeCbl and AdoCbl were expressed as% of total radioactivity of all cobalamin derivates including cyanocobalamin and hydroxocobalymin.

### *In silico* predictions

Putative splicing regulatory elements were predicted using the ESEfinder 3.0 program (http://rulai.cshl.edu/cgi-bin/tools/ESE3/esefinder.cgi?process=home) (36).

### *In silico* analysis of RNA-seq data

Geuvadis BAM files containing read alignments were downloaded and a MISO compatible annotation of the *MTRR* pseudoexon was generated using an in-house Perl script. MISO was run and the resulting estimates were filtered so that only samples with at least one read supporting the inclusion product were used to estimate the overall inclusion level. MISO estimates of pseudoexon inclusion were used as input to the groupBy program from the BEDTools toolkit (37) to estimate mean inclusion and standard deviation, which were used to calculate the confidence interval.

## RESULTS

### *MTRR* pseudoexon inclusion depends on the balanced interplay between ESS and ESEs elements

**Figure 1.**
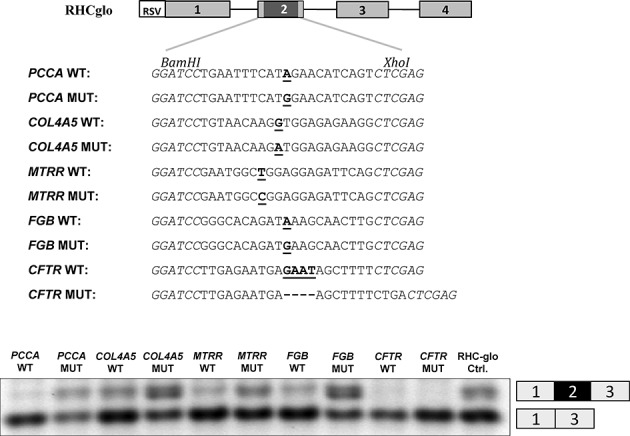
RHC-Glo splicing reporter minigene assay. Upper panel: schematic representation of the RHC-Glo splicing reporter minigene and the sub-cloned sequences. Lower panel: Agarose gel electrophoresis of RT-PCR minigene splicing products expressed in HEK293 cells. Inclusion or exclusion of exon 2 is indicated on the right. The gel pictures are representative results from two experiments.

We have previously shown that the c.903+469T>C mutation in the *MTRR* gene strengthens a preexisting SRSF1-binding motif and thus that pseudoexon activation is most likely caused by creation of an ESE and not loss of an ESS (18). In order to further examine the activity of this motif we inserted wild-type and mutant sequences from the *MTRR* pseudoexon and other pseudoexons where mutations outside the splice site sequences cause pseudoexon activation (20,22–24) into the RHC-Glo splicing reporter minigene (32) and transfected HEK293 cells (Figure 1). Transcripts from the wild-type minigenes show no inclusion of the alternatively spliced exon 2, whereas all the mutant minigenes, except the one harboring the mutant *CFTR* sequence, includes the test exon. This indicates that for all the tested pseudoexon sequences, except *CFTR*, the disease-causing mutation causes pseudoexon inclusion by creating/strengthening an ESE which is able to drive exon inclusion in another genomic context. This further confirms that the c.903+469T>C mutation creates an ESE and it is also consistent with the proposed mechanisms, namely ESE creation, for the other tested pseudoexons. The observed lack of reporter exon inclusion from the mutant *CFTR* sequence is consistent with the proposed mechanism of loss of an hnRNP A2 binding ESS by the mutation (24) and suggests that the mutant *CFTR* sequence does not have ESE activity.

The level and activity of splicing regulatory factors, like SRSF1, vary between cell types. Therefore, we determined whether *MTRR* pseudoexon activation also varies between different cell-types. For this analysis we transiently transfected wild-type and mutant *MTRR*-minigenes (18) into HEK293, HeLa and HepG2 cells (Supplementary Figure S1). RT-PCR analysis shows that pseudoexon inclusion from the mutant minigene is most efficient in HEK293 cells, nearly no pseudoexon inclusion is observed in HeLa cells and some pseudoexon inclusion from the mutant minigene is observed in HepG2 cells. These results demonstrate that activation of the mutant *MTRR* pseudoexon is influenced by cell-type, which in turn suggests that the degree of missplicing and local enzyme deficiency may vary between different tissues.

**Figure 2.**
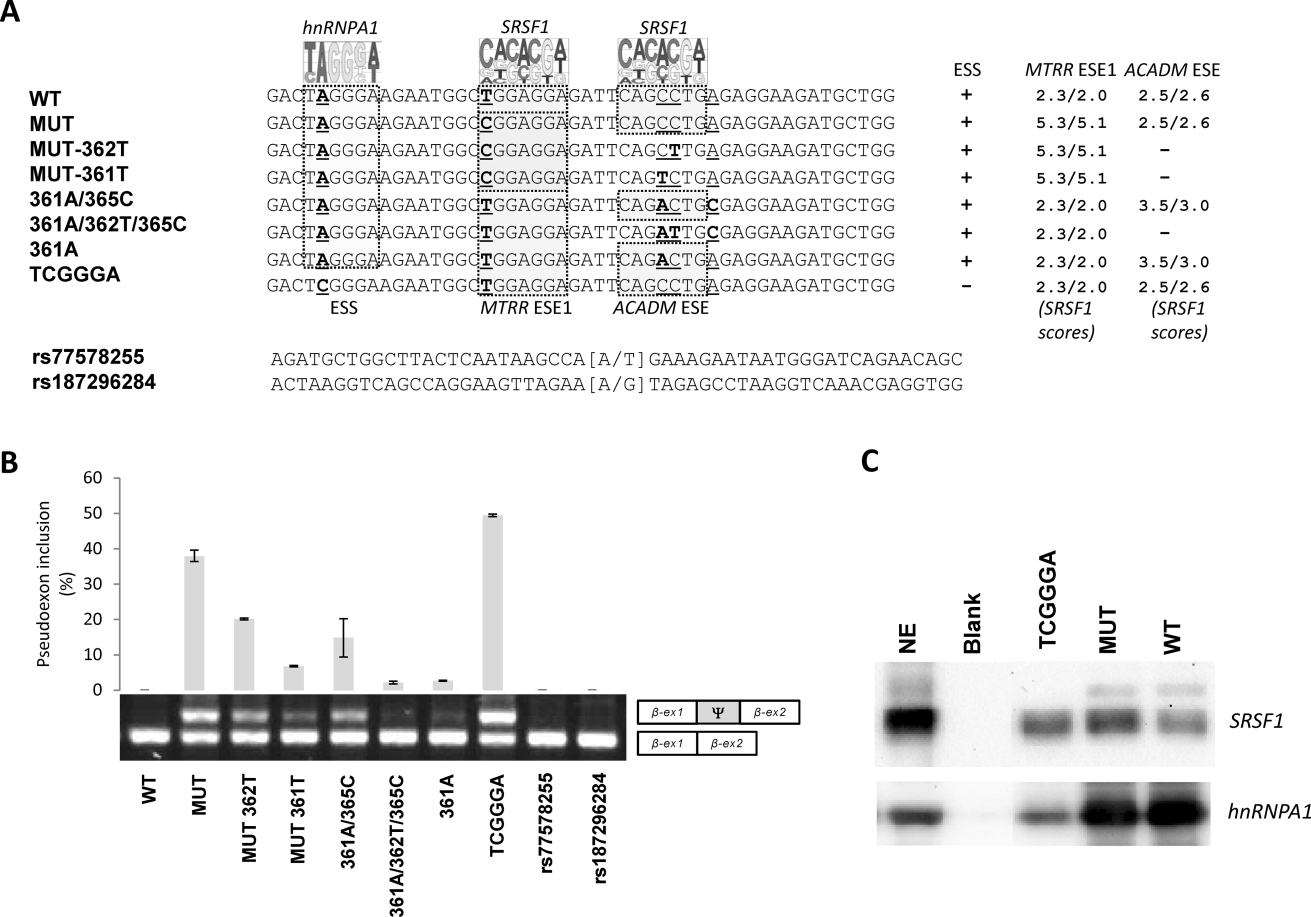
The balanced interplay between an hnRNP A1 binding ESS and two SRSF1 binding ESEs dictate *MTRR* pseudoexon activation. (**A**) Schematic representation of the sequences from *MTRR*-minigenes used and the nucleotide changes introduced. A pictogram of hnRNP A1 position weight matrix (38) is shown above the putative ESS and the invariant position 2 of this motif is underscored. A pictogram of the SRSF1 position weight matrix (36) is shown above the previously described ESE1 (18) and the *ACADM* like ESE. Indicated positions 361, 362 and 365 are relative to the *ACADM* coding sequence. Nucleotide positions that were changed are underscored. (**B**) Splicing minigene assay. *MTRR*-minigenes were transiently transfected into HEK293. After RNA isolation the splicing products were analyzed by RT-PCR. The upper panel shows the average of pseudoexon inclusion from two measurements of each duplicate. Error bars represent the range. Quantification of PCR products was performed using a Fragment Analyzer instrument. The lower panel shows a sample agarose gel electrophoresis displaying pseudoexon inclusion levels in the different cell lines. The lower bands represent correctly spliced exons, whereas the upper bands represent *MTRR* pseudoexon inserted between minigene exons. Ψ marks the pseudoexon. (**C**) Binding of hnRNPA1 and SRSF1 proteins. Biotinylated RNA oligonucleotides were used in a pull-down experiment with HeLa nuclear extract followed by SDS PAGE and western blot analysis using antibodies against SRSF1 and hnRNP A1. Blank indicates a control lane from pull down without RNA oligonucleotides. NE is nuclear extract. The displayed blots are representative result from at least three pull-down experiments.

Because exon recognition is usually determined by a finely tuned balance between positive and negative splicing regulatory sequences, we hypothesized that additional SREs may contribute to the regulation of *MTRR* pseudoexon splicing. Therefore we inspected the sequences flanking the ESE created by the c.903+469T>C mutation. A potential SRSF1 binding motif (CAGCCTG; Figure 2A) similar to the SRSF1-binding ESE (CAGACTG) present in *ACADM* exon 5 (33) is present 11 bp downstream from the created ESE and a hnRNP A1 binding motif (38) recently demonstrated to cause exon skipping and disease when it is created by a mutation in the weak exon 2 in *ETFDH* (13), is present 9 bp upstream (Figure 2A). To investigate the potential role of these motifs in regulation of pseudoexon inclusion, we designed a set of *MTRR* minigenes where the *ACADM*-like motif is disrupted in a mutant *MTRR* background (MUT-362T and MUT-361T) and the hnRNP A1 motif is disrupted in the wild-type *MTRR* background (WT-TCGGGA) (Figure 2A). Furthermore, we generated the original *ACADM* ESE (WT-361A/365C) and its corresponding ESE-inactivating c.362C>T mutant version in the wild-type *MTRR* minigene (WT-361A/362T/365C). Disruption of the *ACADM*-like ESE by introducing the c.362C>T mutation or another ESE-inactivating mutation, c.361A>T, causes decreased pseudoexon inclusion from the minigenes with the activating *MTRR* c.903+469T mutation (Figure 2B).

The native *ACADM* ESE (33) has an adenosine (A) at position c.361, which matches the consensus SRSF1 binding motif (ESE finder score 3.5/3.0) rather than the cytosine (C) (ESE finder score 2.5/2.6) present in the *MTRR* pseudoexon. We speculated whether increasing the SRSF1 score and thus the ESE strength of the *ACADM*-like ESE by changing this nucleotide in the *MTRR* wild-type context would be sufficient to activate pseudoexon inclusion. However, this results only in a very modest increase in pseudoexon inclusion, showing that in the *MTRR* context the C>A change at the position equivalent to *ACADM* c.361 does not increase ESE strength significantly. On the other hand, reconstitution of the full *ACADM* ESE motif including c.365C in the *MTRR* wild-type context shows that this is sufficient to activate pseudoexon inclusion significantly, although this change is apparently located outside the core SRSF1 motif (Figure 2B).

Taken together these data show that the *ACADM*-like motif in the *MTRR* pseudoexon has ESE activity, and that the high levels of *MTRR* pseudoexon inclusion may be accomplished through the synergistic effect of the existing *ACADM*-like ESE and the ESE created by the c.903+469T>C mutation. Moreover, strengthening of either ESE is sufficient to activate pseudoexon inclusion.

Interestingly, also disruption of the hnRNP A1 motif in a wild-type *MTRR* background results in a higher level of pseudoexon activation than the c.903+469T>C mutation, suggesting that inclusion of the pseudoexon is normally repressed by this hnRNP A1 binding ESS motif and further underscoring that mutations in different splicing regulatory elements can activate the pseudoexon. Using RNA-affinity purification we could directly demonstrate that hnRNP A1, as expected, binds to the TAGGGA motif and that this binding is dramatically reduced when the motif is mutated to TCGGGA (Figure 2C). This supports the notion that the increased pseudoexon inclusion from the TCGGGA mutant minigene is due to reduced binding of hnRNPA1 to the ESS motif. Preliminary data employing siRNA mediated knock down of hnRNPA1 in HeLa cells corroborates this notion and indicate that pseudoexon inclusion efficiency increases by a factor of 2–3x (data not shown).

Our results clearly show that *MTRR* pseudoexon activation can result from mutations in several distinct splicing regulatory elements. In line with this, we speculated that also Single Nucleotide Polymorphisms (SNP) reported in this part of *MTRR* could activate the pseudoexon and thereby compromise normal *MTRR* splicing. Consequently, we tested the most common SNPs, rs77578255 (minor allele frequency: A = 0.074) and rs187296284 (minor allele frequency: G = 0.004) for their effect on *MTRR* pseudoexon inclusion. However, these variants did not change the splicing pattern of the *MTRR* minigene and it is thus not likely that they predispose to disease by increasing pseudoexon inclusion efficiency (Figure 2B).

### The SSO complementary to the ESE is most effective in abrogating the *MTRR* pseudoexon activation

**Figure 3.**
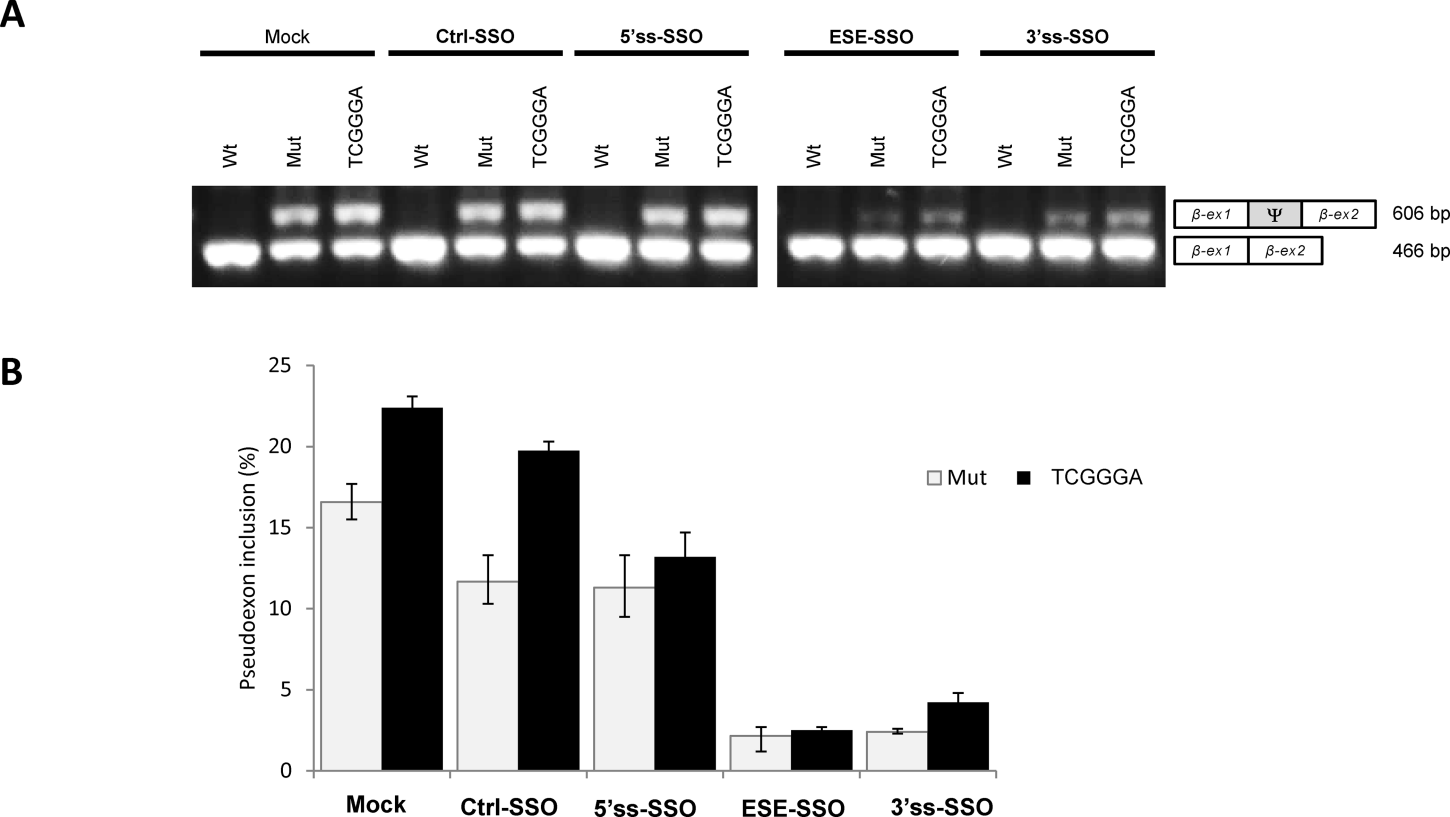
SSO treatment in HEK293 cells transfected with wild-type, mutant and TCGGGA *MTRR*-minigenes. (**A**) Splicing minigene assay. *MTRR*-minigenes were transiently transfected into HEK293. 24 h after the cells were transfected with SSOs targeting either the 5′ss, 3′ss or both ESEs. After RNA isolation the splicing products were analyzed by RT-PCR. The upper panel shows a sample agarose gel electrophoresis displaying pseudoexon inclusion levels. The lower bands represent correctly spliced exons, whereas the upper bands represent *MTRR* pseudoexon inclusion. Ψ marks the pseudoexon. (**B**) Quantification of pseudoexon inclusion. Error bars represent the range. Quantification of PCR products was performed using a Fragment Analyzer instrument.

We next hypothesized that aberrant inclusion of the *MTRR* pseudoexon could be prevented by using SSOs to block binding of splicing factors to the splicing regulatory sequences. We designed three different SSOs, either complementary to the 5′ss, the 3′ss, or overlapping both the ESEs. Minigenes with either the wild-type or the c.903+469T>C mutant *MTRR* pseudoexon, as well as wild-type constructs with a disrupted hnRNP A1 ESS motif (WT-TCGGGA) were transfected into HEK293 cells and treated with SSOs. Treatment with the 3′ss-SSO which blocks the relatively strong 3′ss and the ESE-SSO which blocks both ESEs results in almost complete splicing correction. In contrast, the 5′ss-SSO which blocks the weak 5′ss does not correct splicing (Figure 3). The splicing correction efficiency is similar for the c.903+469T>C mutant minigene and the ESS inactivated WT-TCGGGA minigene. These results indicate that the ESE-SSO, which overlaps both ESEs is very effective in modulating pseudoexon inclusion, consistent with the hypothesis that the c.903+469T>C mutant creates a fundamental ESE and that the *ACADM*-like ESE is a prerequisite for pseudoexon activation.

**Figure 4.**
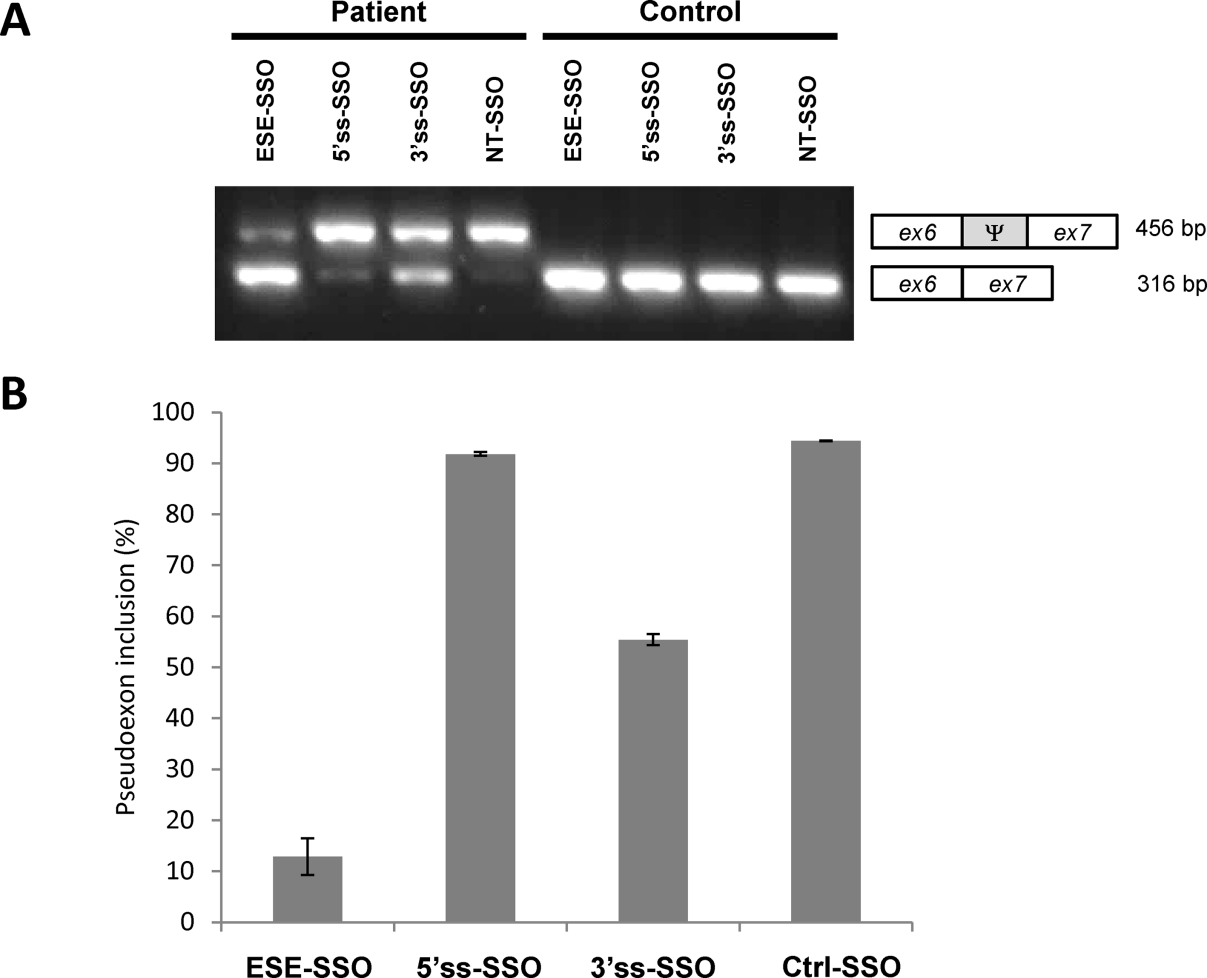
SSO treatment restores *MTRR* splicing in patient cells. (**A**) Fibroblasts from a patient harboring the c.903+469T>C mutation were transfected with SSOs that target either the 5′ss, the 3′ss, both ESEs (ESE-SSO), or a non-targeting sequence (NT). Control fibroblasts were treated in parallel. RNA was extracted after 48 h. A representative agarose gel electrophoresis of the RT-PCR products is shown. The lower bands represent correctly spliced exons, whereas the upper bands represent *MTRR* pseudoexon inclusion between exon 6 and exon 7. Ψ marks the pseudoexon. (**B**) Quantification of pseudoexon inclusion in patient fibroblasts following SSO treatment. Error bars represent the range. Quantification of PCR products was performed using a Fragment Analyzer instrument.

We then examined if SSO treatment could also correct splicing in *MTRR* patient cells, and found that the ESE-SSO effectively induces pseudoexon skipping in patient fibroblasts, restoring correctly spliced *MTRR* mRNA levels almost to those observed in control fibroblast cells (Figure 4). This is consistent with the results from ESE-SSO splicing correction of the minigenes. This effect persists at least until 96 h after treatment and can be achieved with different SSO-doses and transfection procedures (Supplementary Figures S2 and S3 and data not shown). Treatment with the 3′ss-SSO is not as efficient and shows only partial restoration of correct splicing in patient cells, and the 5′splice site targeting 5′ss-SSO does not prevent pseudoexon inclusion in patient fibroblasts (Figure 4).

### ESE-SSO treatment restores *MTRR* enzymatic activity in patient cells

**Figure 5.**
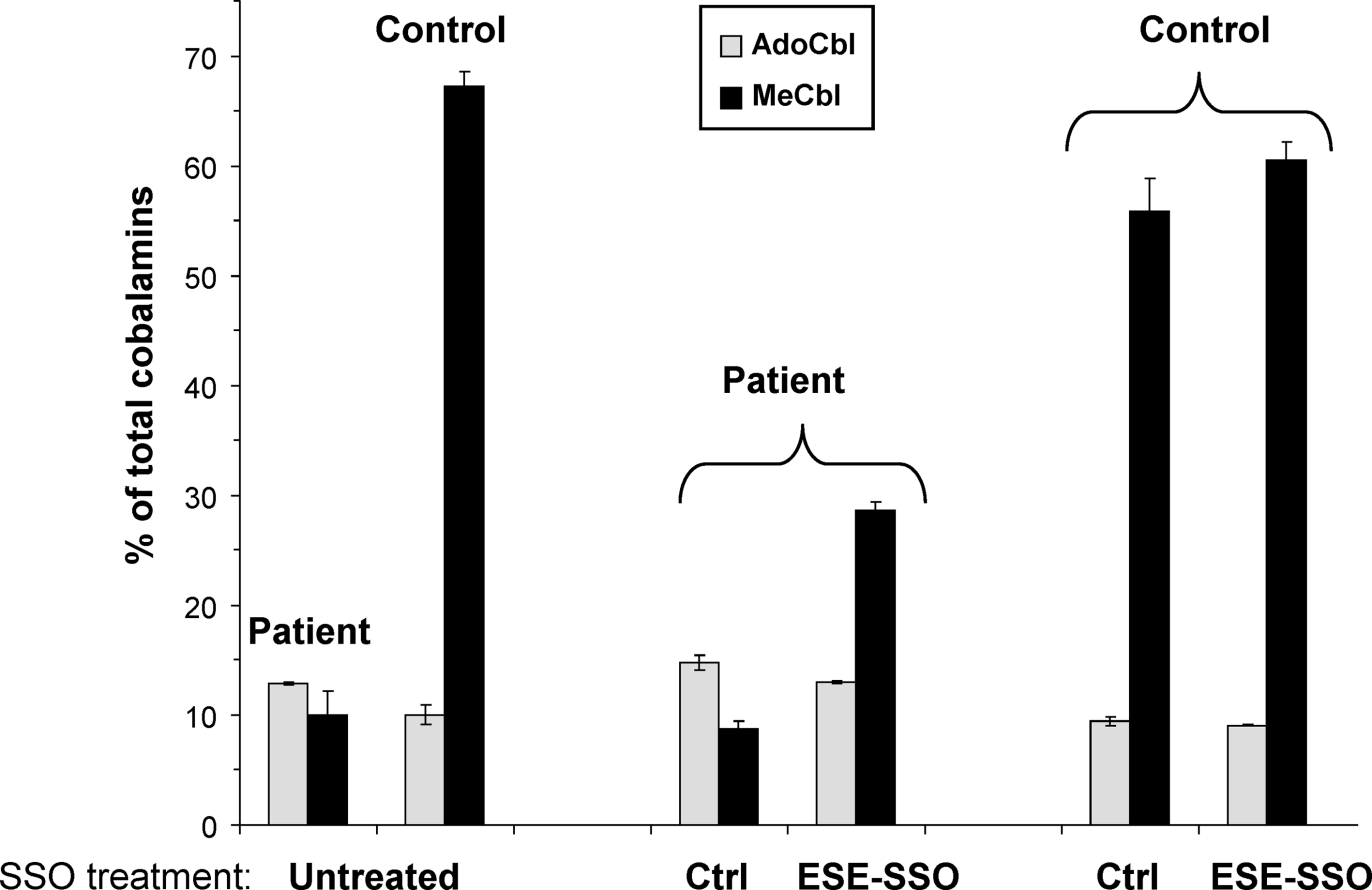
SSO treatment partially restores enzymatic activity of methionine synthase reductase (MTRR). Fibroblasts from a patient homozygous for the c.903+469T>C mutation were transfected with an SSO that target both ESEs (ESE-SSO) or a non-targeting sequence (Ctr). Fibroblasts were then labeled with [^57^Co]cyanocabalamine and MTRR activity was determined indirectly by measuring methylcobalamine (MeCbl) synthesis (expressed as% of total labeled cobalamin derivatives). Adenosylcobalamine (AdoCbl) synthesis was measured as a control. The vertical lines represent the range of duplicate determinations in a representative experiment.

We have shown previously that inclusion of the pseudoexon in the *MTRR* mRNA disrupts the reading frame and creates a premature termination codon, which may lead to decreased mRNA levels due to nonsense-mediated decay (NMD), and consequently results in very low amounts of MTRR enzyme (28). In order to determine whether the observed correction of splicing by ESE-SSO treatment (Figure 4) results in restoration of MTRR activity we measured methylcobolamine (MeCbl) synthesis in patient and control fibroblasts after ESE-SSO treatment and monitored adenosylcobolamine (AdoCbl) synthesis as a control. For this purpose, patient and control fibroblast cells were transfected with ESE-SSO or the non-targeting control SSO by lipofection or electroporation. The transfected cells were then metabolically labeled with [^57^Co]cyanocobalamin and the cobalamin coenzyme synthesis was determined (34). Transfection of patient cells with the ESE-SSO using lipofection resulted in a 3.3-fold increase of MeCbl synthesis and increase of MTRR activity from 16% to 47% (Figure 5). Similar results were obtained when cells were transfected by electroporation (data not shown). Together, these data show that it is possible to rescue MTRR function in patient cells by increasing its activity to approximately 50% of that in controls when the ESE-SSO is used to correct *MTRR* splicing.

## DISCUSSION

Pseudoexon activation was originally thought to be a rare and rather exotic event, which in order to occur requires that the function of more than one splicing regulatory element is altered by mutations (14). Despite this, and despite the fact that analysis of the complete intronic sequences is usually not routine in most diagnostic settings, activation of pseudoexons by single intronic mutations is now frequently reported as disease mechanism (3,17). Although pseudoexon activation in the majority of reported cases is caused by mutations which increase splice site strength, there are also several examples, like the *MTRR* pseudoexon (18), where the activating mutation is located outside the splice sites (19–25). This shows that a group of pseudoexons only require mutation of a single splicing regulatory element located outside the splice sites in order to be fully activated.

In the present study we first show that the point mutations that cause this type of activation of the *Col4A5-*, *FGB-* and *PCCA-*pseudoexons, as well as the *MTRR* pseudoexon, all create ESE elements, which are able to activate splicing in a different genomic context like the RHC-Glo splicing reporter minigene. Our results are consistent with previous studies, which indicate that activation of the *FGB* pseudoexon is caused by creation of an SRSF1 binding ESE (24). They are also consistent with our own previous data (18) showing that activation of the *MTRR* pseudoexon by the c.903+469T>C mutation is due to creation of a strong SRSF1 binding motif, which is identical to a fundamental SRSF1 binding ESE in the RON gene (*MST1R*) (39,40). These data point to ESE activation as a common mechanism for pseudoexon inclusion and they support the notion that the splicing regulatory elements affected by pseudoexon activating mutations are not unique, but represent general splicing regulatory elements.

On this basis, and because splicing regulation is usually regulated by a finely tuned balance between several positive and negative splicing regulatory elements, we hypothesized that also other splicing regulatory elements than the ESE created by the c.903+469T>C mutation are fundamental in regulation of *MTRR* pseudoexon splicing. Consequently, mutations in several parts of this pseudoexon could potentially lead to its inclusion.

### Splicing of the *MTRR* pseudoexon is regulated by several SRE motifs

By examining the pseudoexon sequence surrounding the ESE created by the c.903+469T>C mutation, we identified an *ACADM*-like ESE, which is fundamental for exon 5 inclusion in the *ACADM* gene (33) and a hnRNP A1 binding ESS motif (Figure 2), previously demonstrated to inhibit exon inclusion, when created by disease-causing point mutations in *ETFDH* (13) or *APC* (41).

In *ACADM* exon 5 a c.362C>T point mutation disrupts the SRSF1 binding ESE resulting in skipping of the exon (33). Given the similarity between the *ACADM* ESE (CAGACTGC) and the *ACADM*-like ESE motif found in the *MTRR* pseudoexon (CAGCCTGA), we hypothesized that this sequence could function as an ESE and contribute to activation of the *MTRR* pseudoexon. Our data confirmed this hypothesis and clearly demonstrated that the *ACADM*-like motif has ESE activity and that this activity could be disrupted in the *MTRR* mutant background by introduction of the disease-causing *ACADM* c.362C>T (33) or an *ACADM* c.361C>T mutation, which also inhibits *ACADM* exon 5 splicing (unpublished data). This shows that the presence of the *ACADM*-like ESE motif makes the *MTRR* pseudoexon sensitive to activating mutations and that activation by the c.903+469T>C mutation is dependent on this pre-existing *ACADM*-like ESE.

We also changed the balance between positive and negative splicing regulatory elements in the *MTRR* pseudoexon by disrupting the identified hnRNP A1 motif in the wild-type background. This resulted in an even more dramatic activation of the *MTRR* pseudoexon than caused by the c.903+469T>C mutation, illustrating that mutations that disrupt ESS sequences can also cause *MTRR* pseudoexon inclusion and that this particular hnRNPA1 binding ESS normally contributes to inhibit pseudoexon inclusion. This is consistent with the idea that pseudoexons are not recognized by the spliceosome because they are enriched with splicing silencer motifs (15).

The fact that both the stimulatory effect of *ACADM* like SRSF1 binding ESE and the inhibitory effect of the hnRNP A1 binding ESS motif previously found in the *ETFDH* and *APC* genes have comparable effects in the *MTRR* pseudoexon further supports the notion that the splicing regulatory elements, which regulate pseudoexon splicing are general and able to function in different genomic contexts.

**Figure 6.**
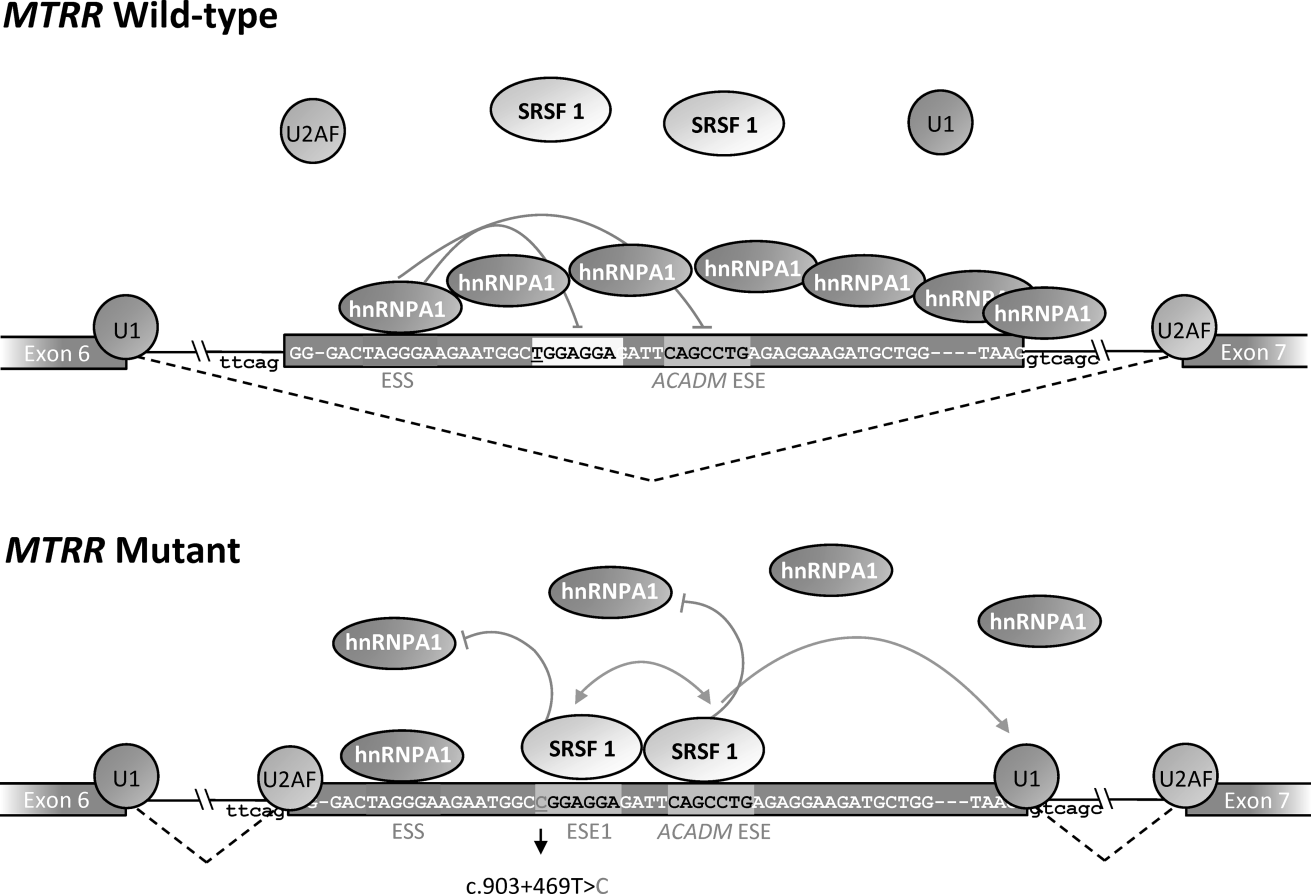
Splicing model of the *MTRR* pseudoexon activation induced by the c.903+469T>C mutation. The wild-type sequence harbors a very weak SRSF1 binding ESE1, a flanking *ACADM* like SRSF1 binding ESE and a hnRNP A1 binding ESS. In the wild-type *MTRR* gene binding of hnRNP A1 to the TAGGGA high affinity ESS inhibits splicing because this initiates cooperative spreading of hnRNPA1 to weaker sites in a 3′-to-5′ direction thereby directly blocking access to the 5′ splice site and other splicing regulatory elements. Simultaneously binding of hnRNP A1 to the ESS inhibits binding of SRSF1 both to the very weak SRSF1 motif in ESE1 and to the *ACADM*-like ESE motif. This also affects recognition of the weak 5′ss by U1 snRNP, since this may be dependent on SRSF1 binding to the ESEs. When the c.903+469T>C mutation is present it changes the weak SRSF1 motif in ESE1 to a very strong site, which may act synergistically with the *ACADM*-like SRSF1 ESE to recruit U1snRNP to the weak 5′ss despite binding of hnRNP A1 to the flanking ESS does not seem to be affected. SRSF1 binding to the ESEs also antagonizes the cooperative spreading of hnRNPA1 binding from the TAGGGA high affinity ESS. Together this leads to pseudoexon activation and the abnormal splicing of the *MTRR* transcript observed in patients.

Taken together, our data support a model where binding of hnRNP A1 to the TAGGGA high affinity ESS in wild-type *MTRR* inhibits splicing, maybe because hnRNP A1 bound to the high affinity site initiates cooperative spreading of hnRNPA1 to weaker sites in a 3′-to-5′ direction thereby directly blocking access to the 5′ splice site and other splicing regulatory elements (42). Simultaneously binding of hnRNP A1 to the ESS inhibits binding of SRSF1 both to the very weak SRSF1 motif in ESE1 in the wild-type pseudoexon and to the *ACADM*-like ESE motif. This may affect recognition of the weak 5′ss by U1 snRNP, since this may be dependent on SRSF1 binding to the ESEs. This model contributes to explain how wild-type pseudoexon recognition and splicing into the *MTRR* mRNA is prevented. When the c.903+469T>C mutation is present, it changes the weak SRSF1 motif in ESE1 to a very strong site, which may act synergistically with the *ACADM*-like SRSF1 ESE to recruit U1 snRNP to the weak 5′ss despite binding of hnRNP A1 to the flanking ESS does not seem to be affected. SRSF1 binding to the ESEs may also antagonize the cooperative spreading of hnRNPA1 binding from the TAGGGA high affinity ESS (Figure 6). Together this leads to pseudoexon activation and the abnormal splicing of the *MTRR* transcript observed in patients. This model is supported by the fact that hnRNP A1 is a well-established antagonist of SRSF1 (43,44). Moreover, we have recently observed a similar type of balanced interplay between hnRNPA1 and SRSF1 in *ETFDH* exon 2, where creation of the hnRNPA1 binding ESS is suggested to cause exon skipping by abolishing binding to flanking SRSF1 sites (13). Interestingly, in *ACADM* exon 5 a balance also exists between the SRSF1 binding ESE, and a flanking hnRNPA1 binding ESS. Here a silent polymorphism in the ESS can abolish hnRNPA1 binding and thereby alleviate the need for the strong SRSF1 binding ESE to be present in order to ensure exon 5 inclusion (33). Finally, in the RON oncogene (*MST1R*), a strong SRSF1 binding ESE with a sequence identical to the *MTRR* pseudoexon ESE created by the c.903+469T>C mutation, is also antagonized by a hnRNPA1 binding silencer (45).

Our data show that pseudoexon activation is a dynamic process, where different mutations may cause different levels of activation dependent on which splicing regulatory element they affect and what the effect on the functionality of that element is. Moreover, by transfection of different cell types we observed cell type specific differences in the level of pseudoexon inclusion. Taken together these data suggested to us that perhaps also the wild-type *MTRR* pseudoexon has a minimal level of inclusion in normal cells, and perhaps the degree of pseudoexon inclusion may vary between individuals.

The Geuvadis RNA sequencing project (46) has made the RNA-seq data of 467 individuals publicly available, and we used the splicing analysis program MISO (47) to quantitate the level of pseudoexon inclusion in these samples. We identified detectable wild-type *MTRR* pseudoexon inclusion (at least one read) in 140 individuals (30%), with a mean estimated inclusion of 4% (Confidence interval (CI-95%) = 3.6%–4.3%). This confirms that the wild-type *MTRR* pseudoexon is indeed included at a minimal level in blood cells from some individuals. Consequently, we extended our analysis to include also the *ATM*, *CFTR*, *COL4A5*, *PCCA*, *FGB, GLA* as well as *MCCC2* and *MFGE8* pseudoexons since these all have functional splice sites and can be activated by a single mutation to cause activation and disease, suggesting that they may also be at the verge of activation and that minor changes, such as genetic variation in splicing regulatory elements or changes in the levels of splicing factors may lead to inclusion. We observed low levels of inclusion of the *PCCA* pseudoexon located in intron 14 (22), which was detected by at least 1 read in 14 samples, with a mean estimated inclusion of 5.0% (CI-95% = 4.3%–5.7%). The *GLA* pseudoexon located in intron 4 (19) was detected by at least 1 read in 286 samples, with a mean estimated inclusion of 6.0% (CI-95% = 5.6%–6.4%). For the *MCCC2* pseudoexon in intron 10 (48), we identified exon inclusion (min 1 read) in 114 samples, with a mean estimated inclusion of 4.5% (CI-95% = 3.5%–5.2%). The pseudoexon reported in intron 6 of *MFGE8* (25) was detected in 44 samples, with a mean estimated inclusion of as much as 14.9% (CI-95% = 12.6%–17.3%). Manual inspection revealed significant intron retention relative to the *MFGE8* pseudoexon inclusion level, likely affecting the MISO analysis. However, in 40 samples we detected at least one splice junction read between the pseudoexon and the neighboring exons, confirming inclusion of the pseudoexon. We could not detect any inclusion of the *ATM*, *CFTR*, *COL4A5* and *FGB* pseudoexons (20–21,23–24) in the Geuvadis sample set, but this was not surprising since these genes are all expressed at low levels in lymphocytes.

However, detection of low levels of the *MTRR*, *GLA, MFGE8, MCCC2* and *PCCA* pseudoexon inclusion in normal lymphocytes by RNA-seq illustrates that pseudoexon inclusion is dynamic, and most importantly, that identification of new pseudoexons using RNA-seq of normal cells is possible. This approach could be used to identify pseudoexons, which are at risk of becoming active by single mutational events or by SNPs. Such knowledge would be valuable for evaluation of disease associated sequence variations located in introns in the future.

### SSOs can efficiently correct splicing of the *MTRR* pseudoexon and increase enzyme activity

SSOs have been used successfully in recent years as a therapeutic approach to correct splicing defects caused by disease-causing mutations in numerous genes (22,24,49–54).

In the present study we tested three different 2′-OMe Phosphorothioate RNA SSOs, targeted to the 5′splice site, the 3′splice site or a region harboring both ESEs, for their ability to correct aberrant splicing caused by the c.903+469T>C mutation. The SSOs targeted to the 3′ss (3′ss-SSO) or the ESEs (ESE-SSO) significantly reduced pseudoexon inclusion, while the SSO complementary to the 5′ss (5′ss-SSO) had no effect (Figure 4). These results could simply be explained by limited binding of the 5′ss-SSO to the weak 5′ splice site, due to limited access to the target sequence during splicing or that the 5′ss-SSO itself forms a secondary structure inhibiting its binding.

Alternatively, the lack of effect from the 5′ss-SSO could be due to the fact that the 5′ss of the *MTRR* pseudoexon is weak and that pseudoexon recognition primarily relies on the ESEs and the strong 3′ss to initiate recruitment of the spliceosomal components. Once spliceosome formation is initiated recruitment of U1 snRNP displaces the 5′ss-SSO at the 5′ss and the pseudoexon can be spliced. Conversely, when the 3′ss-SSO blocks the 3′ss or the ESE-SSO blocks the ESE motifs, recruitment of the spliceosome is inhibited. The 5′ss is too weak to initiate recruitment of spliceosomal components and the pseudoexon is instead skipped. If this hypothesis is correct it might imply that SSOs targeting the strongest splice site or a strong ESE would be most efficient in inhibiting inclusion of activated pseudoexons. Although no systematic testing of this hypothesis has been performed it is supported by a recent study reporting SSO mediated inhibition of an activated pseudoexon in the *CAPN3* gene (54). In this case the disease causing mutation activates the pseudoexon by increasing the strength of a weak 3′splice site. However, recognition of the activated mutant pseudoexon is dependent on an SRSF1 binding ESE. Targeting the strengthened 3′splice site with an SSO only resulted in partial pseudoexon skipping, whereas targeting the strong 5′splice site or the SRSF1 ESE rescued splicing almost completely. The efficiency of SSO mediated ESE blocking to achieve skipping of activated pseudoexons was also demonstrated for the FGB pseudoexon. Here an SSO which targeted to the SRSF1 binding ESE created by the disease-causing mutation in the FGB pseudoexon also very efficiently inhibited pseudoexon inclusion from an *FGB* splicing reporter minigene (24).

Interestingly, treatment with our ESE-SSO could also normalize splicing from the *MTRR* TCGGGA minigene, where pseudoexon activation is caused by loss of the hnRNPA1 binding ESS. First of all, this underscores that the function of the *ACADM*-like ESE is critical for *MTRR* pseudoexon activation, since the ESE-SSO blocking of this ESE, in the absence of the hnRNP A1 binding ESS, is sufficient to inactivate pseudoexon inclusion. Secondly, it illustrates that SSO blocking of a critical ESE is sufficient to counteract pseudoexon activation caused by mutations affecting other critical elements in the pseudoexon. This suggests that optimizing an SSO-based therapy targeting only one critical ESE in a pseudoexon could be used to antagonize the activating effect of several different activating mutations located in other parts of the pseudoexon thereby alleviating the need for designing specific SSOs targeting each new activating mutation. Moreover, the risk of undesired splicing changes in other genes resulting from off-target effects of the SSO is limited when targeting exonic sequences harboring ESEs, since such sequences, in contrast to splice site sequences, are gene-specific.

Finally, we show that SSO therapy targeting the ESE is able to correct abnormal splicing in patient cells harboring the c.903+469T>C mutation and that this increases MTRR enzymatic activity to levels which could be sufficient to obtain therapeutic effect.

The present study shows that SSO blocking of ESEs could be a promising strategy to treat the increasing number of activated pseudoexons, which are likely to be detected as this phenomenon is receiving more attention and high throughput sequencing techniques are adopted into clinical settings.

## SUPPLEMENTARY DATA

Supplementary Data are available at NAR Online.

SUPPLEMENTARY DATA
