# Supplementary material for: Splice-shifting oligonucleotide (SSO) mediated blocking of an exonic splicing enhancer (ESE) created by the prevalent c.903+469T>C MTRR mutation corrects splicing and restores enzyme activity in patient cells
Source: Nucleic Acids Res. 2015 Apr 15;43(9):4627–39. doi: 10.1093/nar/gkv275 (PMC4482064; doi:10.1093/nar/gkv275)
Supplement: SUPPLEMENTARY DATA [file supp_43_9_4627__index.html]

Splice-shifting oligonucleotide (SSO) mediated blocking of an exonic splicing enhancer (ESE) created by the prevalent c.903+469T>C MTRR mutation corrects splicing and restores enzyme activity in patient cells — SUPPLEMENTARY DATA 

# Splice-shifting oligonucleotide (SSO) mediated blocking of an exonic splicing enhancer (ESE) created by the prevalent c.903+469T>C *MTRR* mutation corrects splicing and restores enzyme activity in patient cells

## SUPPLEMENTARY DATA

**Files in this Data Supplement:**

- SUPPLEMENTARY FIGURES
