# Supplementary material for: Splice-shifting oligonucleotide (SSO) mediated blocking of an exonic splicing enhancer (ESE) created by the prevalent c.903+469T>C MTRR mutation corrects splicing and restores enzyme activity in patient cells
Source: Nucleic Acids Res. 2015 Apr 15;43(9):4627–39. doi: 10.1093/nar/gkv275 (PMC4482064; doi:10.1093/nar/gkv275)
Supplement: SUPPLEMENTARY DATA [file supp_gkv275_nar-00541-y-2015-File008.docx]

**
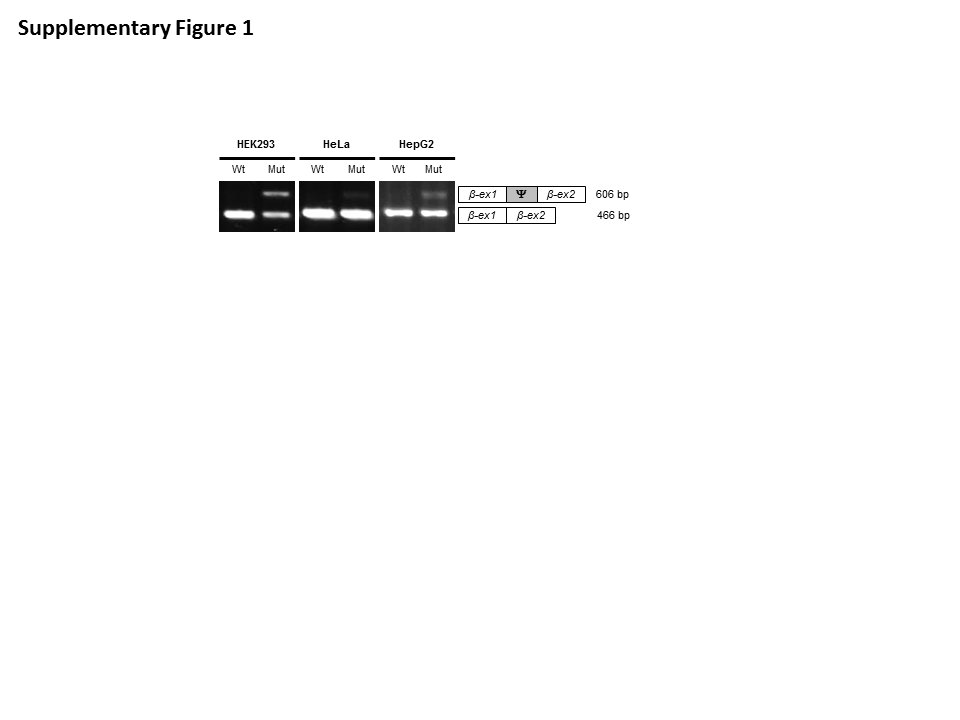
**

**Supplementary Figure 1.** *MTRR* pseudoexon activation is cell type dependent. β-globin minigenes harboring either the wild-type or mutant *MTRR* pseudoexon were transiently transfected into HEK293, HeLa and HepG2 cells. After RNA isolation the splicing products were analyzed by RT-PCR. A representative agarose gel electrophoresis is shown displaying pseudoexon inclusion levels in the different cell lines. The lower bands represent correctly spliced exons, whereas the upper bands represent *MTRR* pseudoexon inserted between minigene exons.


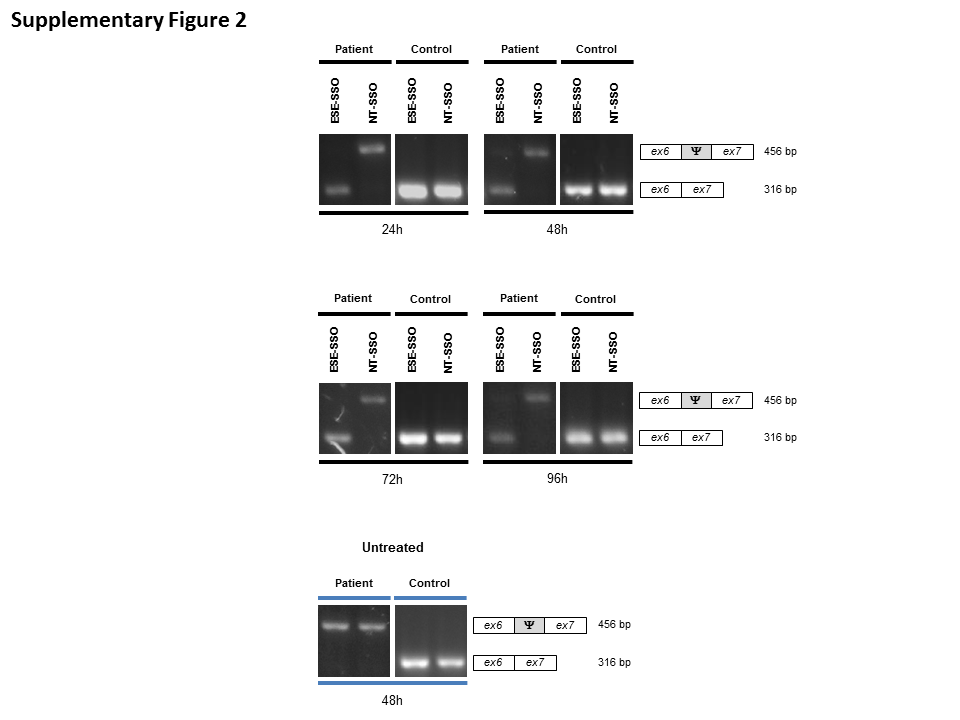


**Supplementary Figure 2.** SSO treatment persists along a time course of 96 hours. Patient fibroblasts harboring the c.903+469T>C mutation were transfected with either, an SSO that targets both ESEs (ESE-SSO) or a non-targeting sequence (NT-SSO). Healthy fibroblasts were treated in parallel as a control. Total RNA was isolated 24, 48, 72 or 96 hours after SSO treatment. At 48 hours, RNA was also extracted from untreated cells as a control. A representative agarose gel electrophoresis of the RT-PCR products is displayed. The lower bands represent correctly spliced exons, whereas the upper bands represent *MTRR* pseudoexon inserted between exon 6 and exon 7. Ψ marks the pseudoexon.


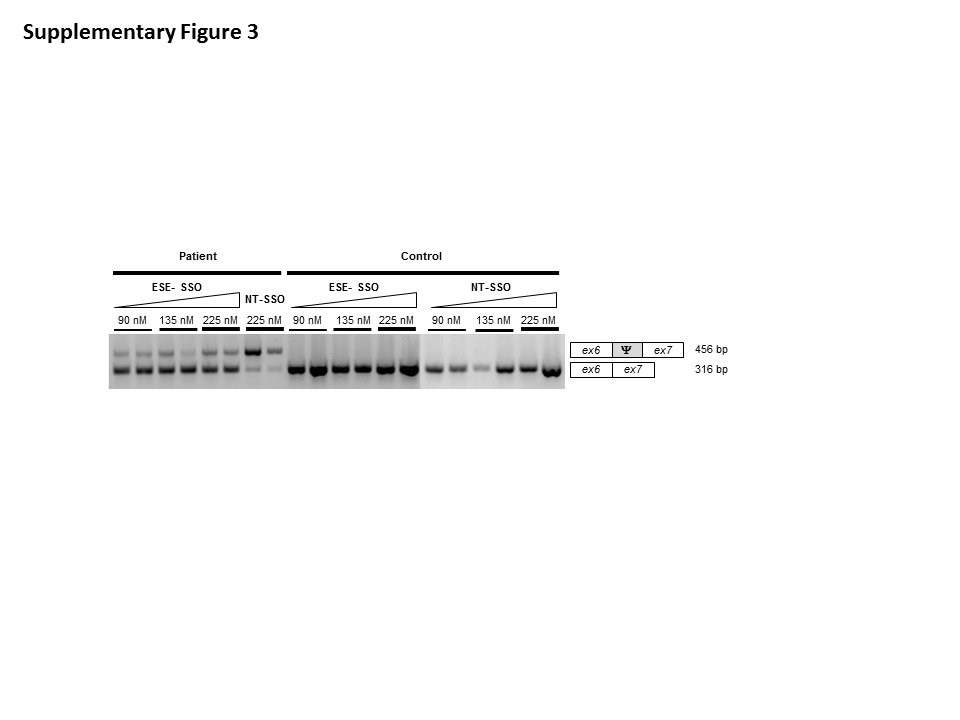


**Supplementary Figure 3.** Dose dependent SSO treatment of patient and control fibroblasts. Patient fibroblasts were transfected with 90 nM, 135 nM or 225 nM of the SSO that targets both ESEs (ESE-SSO) and 225 nM of a non-targeting sequence (NT-SSO). As a control healthy fibroblasts were treated with 90 nM, 135 nM or 225 nM of either the ESE-SSO or the NT-SSO. A representative agarose gel electrophoresis of the RT-PCR products is shown. The lower bands represent correctly spliced exons, whereas the upper bands represent *MTRR* pseudoexon inserted between exon 6 and exon 7. Ψ marks the pseudoexon.
